# Epitranscriptomics: A New Regulatory Mechanism of Brain Development and Function

**DOI:** 10.3389/fnins.2018.00085

**Published:** 2018-02-20

**Authors:** Florian Noack, Federico Calegari

**Affiliations:** DFG–Research Center and Cluster of Excellence for Regenerative Therapies, Dresden, Germany

**Keywords:** epitranscriptomics, RNA-epigenetics, epitranscriptome-editing, N6-methyladenosine, 5-methylcytosine, 5-hydroxymethylcytosine, neural stem cells, brain development

## Abstract

Epigenetic modifications of DNA and chromatin are long known to control stem cell differentiation and organ function but the role of similar modifications at the level or regulatory RNAs is just beginning to emerge. Over 160 RNA modifications have been identified but their abundance, distribution and functional significance are not known. The few available maps of RNA modifications indicated their dynamic regulation during somatic stem cell differentiation, brain development and function in adulthood suggesting a hitherto unsuspected layer of regulation both at the level of RNA metabolism and post-transcriptional control of gene expression. The advent of programmable, RNA-specific CRISPR-Cas editing platforms together with the identification of RNA modifying enzymes now offers the opportunity to investigate the functional role of these elusive epitranscriptome changes. Here, we discuss recent insights in studying the most abundant modifications in functional mRNAs and lncRNAs, N6-methyladenosine and 5-(hydroxy-)methylcytosine, and their role in regulating somatic stem cell differentiation with particular attention to neural stem cells during mammalian corticogenesis. An outlook on novel CRISPR-Cas based systems that allow stem cell reprogramming by epitranscriptome-editing will also be discussed.

## Introduction

During embryonic development, rapid changes in protein expression and their activity are required to initiate and promote the switch from proliferation to differentiation of stem cells. Historically, stem cell research has been primarily focused on understanding the control of gene expression at the transcriptional level by transcription factors or epigenetic modifications of DNA or histones (Atlasi and Stunnenberg, 2017). In addition, post-translational modifications are long known to influence protein stability and activity, which by definition has implications in all biological processes including in controlling the proliferation versus differentiation of somatic stem cells during development and adulthood. While modifications of both DNA and proteins have long been the focus of intensive research, very little is known about the modifications that may occur at the level of the molecules that transduce the genetic message from the DNA to the proteins: functional mRNAs.

Overall, mRNAs and protein levels fairly correlate but about half of the variation in the latter cannot be explained by mRNA concentrations alone (Vogel and Marcotte, 2012) implying that post-transcriptional regulation must also play critical roles in controlling protein abundance. For instance, many aspects of mRNA metabolisms including, among others, splicing, capping, polyadenylation, nuclear export, and rates of translation versus degradation are regulated during brain development by RNA-binding proteins and/or microRNAs (Lennox et al., 2018). In addition to these classical mechanisms for post-transcriptional control of protein expression, over 150 chemical modification of nucleotides are being listed in a recently developed online database of RNA modifications (Boccaletto et al., 2017). However the abundance, distribution and function of essentially all of these RNA modifications have remained elusive.

Systematic mapping of RNA modifications across the transcriptome of different species and tissues by antibody pull-down or chemical labeling coupled to sequencing have just begun. These efforts revealed that RNA modifications are not only abundant in housekeeping, non-coding RNAs, such as tRNAs and rRNAs (He, 2010), but are also commonly found within functional mRNAs and lncRNAs (Boccaletto et al., 2017). Interestingly, some of the mapped modifications showed very dynamic patterns and tissue-specific distribution supporting the notion that they may harbor regulatory potential comparable to that of classical epigenetic marks, thus, opening up the new field of RNA-epigenetics (He, 2010) or epitranscriptomics (Saletore et al., 2012).

This field is still in its infancy and mapping the vast majority of the many RNA modifications is highly problematic due to the need of specific antibodies while lacking the possibility to validate any outcome by alternative methods. This can lead to contradicting results as for example in the case of N1-methyladenosine (m1A). Mapping of m1A by antibody pull-down and sequencing initially led to the conclusion that this modification is broadly abundant within mRNAs (Dominissini et al., 2016; Li et al., 2016), which was later confirmed by methods providing single-nucleotide resolution of m1A modifications (Li et al., 2017b). However, these results were contradicted by another study using a similar experimental approach but showing that m1A at mRNAs is rare and almost exclusively occurring within stem loops equivalent to those of tRNAs and that for this reason might be spuriously introduced by the tRNA m1A-methylation machinery (Safra et al., 2017).

Nevertheless, the rapidly advancing methodologies to characterize the epitranscriptome and the limited number of studies mapping these modifications within functional mRNAs and lncRNAs makes this a fast evolving field. Therefore, in this minireview we will only focus on the three most reproducibly mapped and intensely studied mRNA modifications known to date: N6-methyladenosine (m6A), 5-methylcytosine (5mC), and 5-hydroxymethylcytosine (5hmC). Their functions in different cell types will be discussed with particular attention to neural stem cell differentiation during mammalian corticogenesis and brain function in adulthood.

## N6-methyladenosine

Methylation of adenine at the 6 position (m6A) is commonly found on DNA of prokaryotes but generally rare in eukaryotes and highly debated in mammals (Luo et al., 2015). In contrast, m6A in mRNAs and lncRNAs is frequently found in both prokaryotes and eukaryotes including mammals from rodents to humans (Desrosiers et al., 1975; Wei et al., 1975).

The synthesis of m6A requires the co-transcriptional addition of the methyl group of S-adenosylmethionine to adenine by the METTL3/METTL14/WTAP complex (Liu et al., 2014; Ping et al., 2014; Schwartz et al., 2014). In this complex, METTL3 exhibits the catalytic activity whereas METTL14 (Wang et al., 2016) and WTAP (Ping et al., 2014) provide the RNA binding scaffold. Additionally, FTO (Jia et al., 2011) and ALKBH5 (Zheng et al., 2013) have been identified as m6A demethylases allowing for a dynamic addition and erasure of this epitranscriptional mark. Specifically, FTO oxidizes m6A to the meta-stable N6-hydroxymethyladenosine and N6-formyladenosine that undergo spontaneously conversion to adenosine (Fu et al., 2013) while ALKBH5 directly catalyzes the demethylation of m6A (Zheng et al., 2013).

**Figure 1:**
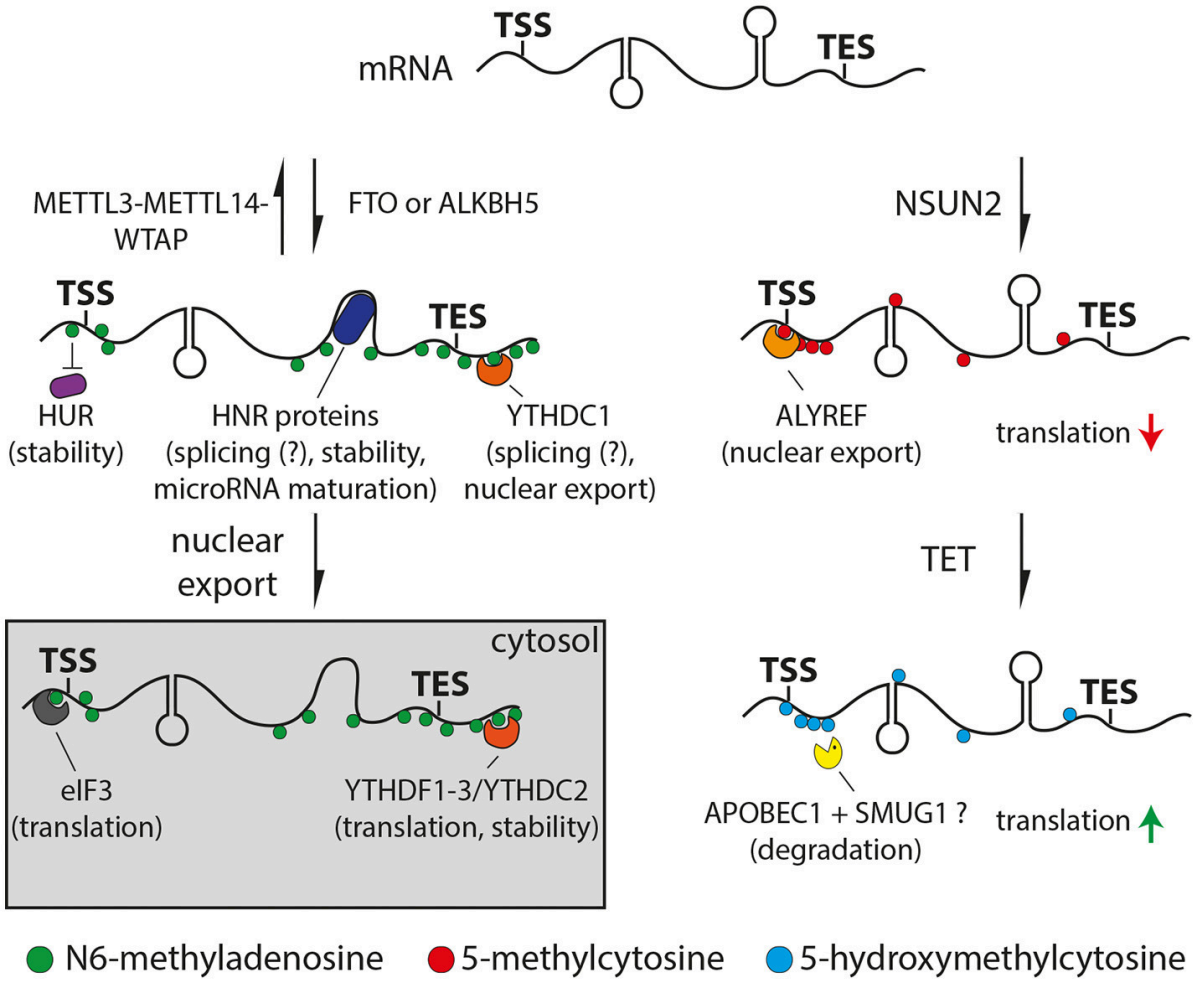
Drawings of N6-methyladenosine (left) or 5-methylcytosine (right) pathways. **Left:** Adenosine is methylated (m6A, green) by the METTL3/METTL14/WTAP complex or removed by the FTO or ALKBH5 demethylases. Proteins can bind m6A directly (YTH and eIF3, orange and gray respectively), indirectly through changes in secondary structure (HNR, dark blue) or be repelled by m6A (HUR, purple). **Right:** Cytosine is methylated at the 5 position (5mC, red) by NSUN2 and oxidized to 5-hydroxymethyl- (5hmC) or 5-formylcytosine (light blue) by TET proteins. 5mC can recruit ALYREF (orange) decreasing translation efficiency, while 5hmC can enhance translation (red and green arrows, respectively). APOBEC1 and SMUG1 (yellow) may be involved in the removal of oxidized 5-methylcytosine resulting in the degradation of the cleaved mRNA. Potential functions of m6A or 5mC readers are indicated in brackets.

Transcriptome-wide mapping of m6A revealed that this modification is mainly deposited at the DRACH (where D = A, G or U; H = A, C or U) consensus motif (Dominissini et al., 2012; Meyer et al., 2012; Schwartz et al., 2014) displaying a conserved pattern across mRNAs and lncRNAs with the highest levels within long exons, transcription end sites, 3′ UTRs (Dominissini et al., 2012; Meyer et al., 2012) and to a lesser extend 5′ UTRs (Meyer et al., 2015; Zhang et al., 2017) (Figure 1, left). Levels of m6A varied across cell types (Chen et al., 2015) and displayed a high evolutionary conservation across mammalian species (Dominissini et al., 2012; Meyer et al., 2012; Batista et al., 2014; Schwartz et al., 2014). Furthermore, m6A levels revealed to be dynamic during embryonic stem cell differentiation (Batista et al., 2014; Schwartz et al., 2014; Chen et al., 2015; Geula et al., 2015) or environmental stimuli such as stress (Dominissini et al., 2012; Zhou et al., 2015). Interestingly, levels of m6A in the brain increase during embryonic and postnatal development and are the highest in the adult brain among all other tissues studied (Meyer et al., 2012).

The molecular function of m6A is just beginning to emerge and is subject of intense research. Several studies indicated roles in controlling various steps of mRNA metabolism including at the level of nuclear export (Zheng et al., 2013; Roundtree et al., 2017), microRNA mediated decay (Meyer et al., 2012), pre-microRNA processing (Alarcón et al., 2015) or polyadenylation (Ke et al., 2015). Furthermore, m6A promotes the binding of YTH or HNRNP protein families to RNA either directly or through m6A-induced changes in the RNA secondary structure, respectively (Figure 1, left) (Dominissini et al., 2012; Liu et al., 2015, 2017). Both YTH and HNRNP proteins are associated with alternative splicing suggesting a functional role of m6A in this process (Dominissini et al., 2012; Liu et al., 2015, 2017; Xiao et al., 2016). Specifically, recent studies suggested that m6A regulates alternative splicing only for a subset of mRNAs and lncRNAs rather than being general unspecific splicing factor (Bartosovic et al., 2017; Ke et al., 2017). Moreover, YTHDF1, 2 and 3 were found to be involved in translation (Meyer et al., 2015; Zhou et al., 2015; Shi et al., 2017; Slobodin et al., 2017) and RNA degradation (Wang et al., 2014a,b; Shi et al., 2017; Zhang et al., 2017) via their combinatorial binding. For example, the binding of YTHDF1 promoted mRNA translation due to the recruitment of the eukaryotic initiation factor 3 (eIF3) (Wang et al., 2015), which can also directly interact with m6A (Figure 1, left) (Meyer et al., 2015). On the other hand, YTHDF2 has been reported to facilitate mRNA decay by recruiting deadenylases (Du et al., 2016). Finally, m6A can also inhibit RNA-protein interactions as shown for the well-established RNA stabilizer HuR, resulting in an increased RNA decay (Figure 1, left) (Wang et al., 2014b). Altogether, m6A can at the same time burst and sharpen the levels of critical proteins by promoting the rate of translation and a faster decay of functional RNAs, respectively. In this context it is interesting to note that transcription factors and genes required for cell-type specific processes show higher levels of m6A compared to housekeeping genes (Batista et al., 2014; Schwartz et al., 2014; Wang et al., 2014b; Chen et al., 2015; Yoon et al., 2017). Therefore, m6A seems to be ideally positioned for playing important roles during cell differentiation by modulating transcriptional networks that swiftly change during fate commitment.

A functional role of m6A in stem cell commitment is further supported by the observation that its ablation, for example by knock-down of METTL3 or METTL14, is compatible with naïve ESC survival but impairs their differentiation due to a higher stability of proliferation and pluripotency factors (Batista et al., 2014; Geula et al., 2015). Conversely, knock-down of ZFP217 led to a higher activity of METTL3, elevated levels of m6A in mRNAs encoding for pluripotency factors and resulting in their lower stability and faster degradation, thus, triggering ESC differentiation (Aguilo et al., 2015). Additionally, overexpression of METTL3 in iPSC promoted reprogramming whereas its knock-down had the opposite effect (Chen et al., 2015).

In animal models, decreasing the levels of m6A by ablation of METTL3 or METTL14 led to defects in (i) sex determination and neuronal function with impaired locomotion in flies (Lence et al., 2016), (ii) morphological and ectoderm and hematopoietic defects in zebrafish (Ping et al., 2014; Zhang et al., 2017) and (iii) embryonic lethality shortly after implantation in mice (Geula et al., 2015).

Moreover, conditional knock-out of METTL14 in mouse embryos resulted in reduced body size and postnatal lethality (Yoon et al., 2017) whereas ablation in the adult brain lead to impaired axonal regeneration (Weng et al., 2018). Concerning neural stem cells during corticogenesis, two recent studies showed that conditional knock-out of METTL14 resulted in aberrant cell cycles, particularly longer S and G2 phases, as well as decreased generation of late-born neurons (Yoon et al., 2017; Wang et al., 2018). While it is unclear whether the causal link between cell cycle length and differentiation (Borrell and Calegari, 2014) applied in this context, these two studies provided different explanations for the observed phenotypes. Yoon et al. reported an impaired differentiation of neural stem cells due to an increased half-life of mRNAs enriched for cell fate determinants and cell cycle regulators upon reduction of m6A suggesting effects on priming and translation of such transcripts (Yoon et al., 2017). On the other hand, Wang et al. showed that the ablation of METTL14 increased differentiation by stabilizing mRNAs for histone modifying enzymes, leading to a decreased neural stem cell pool (Wang et al., 2018). In addition, it is reasonable to expect that also lncRNAs that are important during corticogenesis (Aprea and Calegari, 2015) were also affected by this reduction of m6A upon METTL14 deletion but lncRNAs were not assessed in neither of the two studies.

Additionally, ablation of the m6A eraser FTO in mice led to an increased level of m6A in a subset of mRNAs (Hess et al., 2013), postnatal growth retardation including microcephaly (Fischer et al., 2009; Li et al., 2017a) and impairments in adult neurogenesis (Li et al., 2017a).

In addition to neural stem cells and brain development, roles for m6A modifications were also found during adulthood in particular related to cognitive function such as learning and memory. For example, manipulating the levels of m6A in mouse resulted in changes in neuronal circuitry and activity (Hess et al., 2013) and while the levels of both m6A and FTO acutely changed in the prefrontal cortex or hippocampus of mice upon learning, ablation of FTO enhanced memory formation and consolidation of contextual fear conditioning (Widagdo et al., 2016; Walters et al., 2017). Interestingly, human mutations in FTO were associated with developmental failures specifically of the central nervous system (Boissel et al., 2009), brain atrophy (Ho et al., 2010) and psychological disorders in adulthood (Hess and Brüning, 2014).

Overall, several studies indicated that m6A plays several roles not only in neural stem cell differentiation during development but also in cognitive function and neurological disorders during adulthood, which is consistent with its effects in controlling the stability and expression of certain specific functional RNAs. Uncovering how this specificity is controlled for some, but not others, mRNAs or lncRNAs will be a challenge of future research.

## 5-methylcytosine and 5-hydroxymethylcytosine

5-methylcytosine (5mC) and its oxidized form 5-hydroxymethylcytosine (5hmC) are widely found in eukaryotic DNA and are associated with transcriptional regulation and DNA stability (Li and Zhang, 2014). Four decades ago, 5mC was also described to occur in RNA (Desrosiers et al., 1975) and later found to be highly abundant particularly in tRNAs and rRNAs (Schaefer et al., 2009).

In mammals, 5mC can be catalyzed by DNMT2 (Goll et al., 2006; Tuorto et al., 2012; Khoddami and Cairns, 2013) and proteins of the NOP2/Sun domain RNA methyltransferase family (NSUN). These enzymes target tRNAs or rRNAs in a non-overlapping manner and levels of 5mC at these housekeeping RNAs is important for their stability, biogenesis and function (Motorin et al., 2010). NSUN2 displayed broader substrate specificity including functional mRNAs and lncRNAs (Squires et al., 2012; Hussain et al., 2013; Khoddami and Cairns, 2013; Yang et al., 2017).

Transcriptome-wide profiling of 5mC by bisulfite conversion-based approaches (Schaefer et al., 2009) revealed a high abundance of 5mC in mRNAs at CG dinucleotides around transcription initiation sites (Figure 1, right) (Squires et al., 2012; Hussain et al., 2013; Khoddami and Cairns, 2013; Yang et al., 2017), which also revealed to be evolutionary conserved (Yang et al., 2017). Additionally, the abundance of 5mC in mRNA was found to vary significantly across tissues and transcripts associated with both common metabolic processes and cell-type specific functions (Amort et al., 2017; Yang et al., 2017).

Loss of function of NSUN2 in mouse and human led to motor, neurodevelopmental and cognitive defects (Abbasi-Moheb et al., 2012; Khan et al., 2012; Martinez et al., 2012; Tuorto et al., 2012; Blanco et al., 2014; Komara et al., 2015; Flores et al., 2016). In particular, molecular analysis revealed that the ablation of NSUN2 in mouse caused an increase in angiogenin-induced cleavage of tRNAs, which resulted in a decreased global protein synthesis causing an inhibition of cell differentiation and migration, particularly in the brain (Tuorto et al., 2012; Blanco et al., 2014; Flores et al., 2016). However, these studies did not address additional mRNA-specific effects of NSUN2 ablation as potentially contributing factors to the observed phenotypes. For example, it has been shown that 5mC is required for ALYREF-mediated nuclear export of mRNAs (Yang et al., 2017) and negatively affects translation (Figure 1, right) (Delatte et al., 2016). Furthermore, 5mC might also play a role in microRNA meditated post-transcriptional regulation (Squires et al., 2012; Yang et al., 2017) although this is currently debated (Amort et al., 2017).

Similar to DNA, 5mC at RNA can be oxidized by enzymes of the ten-eleven translocator family (TET) to 5hmC (Fu et al., 2014) and further oxidized to 5-formylcytosine (Huber et al., 2015) and 5-carboxylcytosine (Figure 1, right) (Basanta-Sanchez et al., 2017). Whether or not this may be followed by the excision of the oxidized methylcytosine in RNA, as it is the case for methylation occurring on DNA, is not known. However, evidence for a potential mechanism comes from the observation that SMUG1, a key component of the base-excision repair machinery, can remove oxidized forms of 5-methyluracil (i.e., thymine) from RNA (Jobert et al., 2013). Given that cytosine to uracil conversions are common in RNA (Harjanto et al., 2016) it is tempting to speculate that a similar conversion of oxidized methylcytosine to oxidized 5-methyluracil may occur that would lead to its excision by SMUG1 and RNA degradation (Figure 1, right).

Transcriptome-wide mapping by antibody pull-down revealed low but significant levels of 5hmC in mRNA (Fu et al., 2014; Huber et al., 2015; Yang et al., 2017). Interestingly, the highest levels were found in the brain relative to other tissues (Fu et al., 2014; Huber et al., 2015; Delatte et al., 2016), a specificity that is reminiscent of 5hmC levels in DNA (Lian et al., 2016). This suggests that the cellular environment or activity of TET enzymes may cause both hyper DNA and RNA hydroxymethylation in the brain compared to other tissues.

Studies addressing the role of 5hmC in mammalian mRNAs are lacking because, contrary to 5mC that is synthetized by mRNA-specific enzymes (NSUN2) that do not target DNA, synthesis of 5hmC is mediated by the very same TET enzymes that promiscuously target both RNA and DNA (Lian et al., 2016). For this reason, studies addressing RNA-specific roles of 5hmC are only available in flies that lack DNA methyltransferases and therefore have negligible levels of both 5mC and 5hmC in DNA while still showing abundant 5hmC in RNA. As the only study available to date showing the RNA-specific effects of TET manipulation, high levels of 5hmC in flies correlated with higher translation efficiency (Figure 1, right) and TET knock-down led to brain malformations in the larva and death during the pupal stage (Delatte et al., 2016). Given the current lack of mRNA-specific enzymes to target mammalian 5hmC, systems are needed that allow to overcome the use of conventional genetic deletion and knock-out lines.

## Epitranscriptome editing

The importance of RNA modifications for developmental processes has just begun to emerge and new studies will soon provide us with additional knowledge about their abundance, specificity and role. As a main limitation in this field, functional characterization of mRNA and lncRNAs modifications are so far restricted to the ablation of the enzymes acting as writers, readers or erasers. This has several intrinsic limitations such as that some of these enzymes are unknown, have overlapping or redundant functions or act on different substrates as shown in the case of TET enzymes. Furthermore, ablation of RNA modifying enzymes would still not resolve site-specific roles of such modifications and their impact on specific transcripts.

These limitations can be overcome by the development of site-specific manipulation of RNA modifications as a means to directly prove their functional implications in a way similar to what shown for recent advances in epigenome editing (Thakore et al., 2016). A potential platform for such approaches is provided by the PUF protein family in which a conserved pumilio homology domain (PUF) targets the protein toward a specific RNA sequence (Zamore et al., 1997; Zhang et al., 1997). Engineering of the PUF domain allowed its retargeting toward any 8 nucleotide sequence (Cheong and Hall, 2006; Dong et al., 2011), which was successfully used to track RNAs in living cells (Ozawa et al., 2007), manipulate alternative splicing (Wang et al., 2009, 2013) or translation (Cooke et al., 2011; Abil et al., 2014) and design costume-made RNA endonucleases (Choudhury et al., 2012). As a major drawback of this approach, the 8 nucleotide recognition sequence is typically too short to ensure transcript specificity and retargeting of the PUF domain is laborious and time consuming.

**Figure 2:**
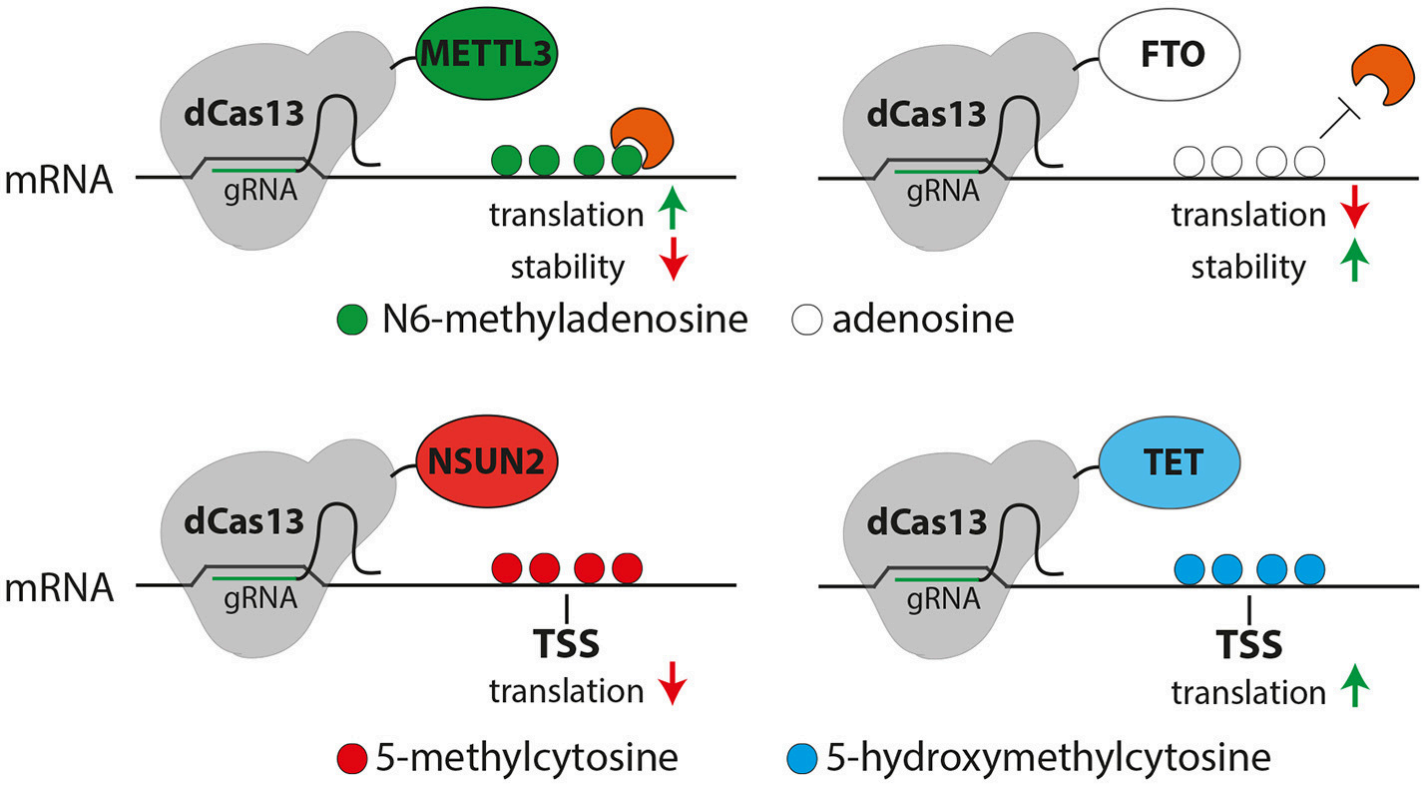
Possible uses of the CRISPR-dCas13 (gray) system for epitranscriptome editing of N6-methyladensosine (m6A, top) or 5-methylcytosine (5mC, bottom). **Top:** Fusing dCas13 together with METTL3 (green) or FTO (white) may allow the site and transcript specific methylation (green) or demethylation (white) of mRNA, respectively resulting in m6A-mediated changes in translation or RNA stability (red or green arrows). **Bottom:** methylation of cytosine (red) or oxidation of 5mC (blue) of cytosine can be triggered by dCas13 fusion to NSUN2 (red) or TET (blue), respectively potentially resulting in a decreased (red arrow, left) or increased (green arrow, right) translation.

These challenges may be overcome by the recently characterized Class 2 subtype VI CRISPR-Cas effector Cas13 that has been used as a programmable endoribonuclease (Abudayyeh et al., 2016, 2017; East-Seletsky et al., 2016; Smargon et al., 2017). Pioneering work by Cox et al. has shown that fusion of a mutant, catalytically inactive Cas13 (dCas13) with the adenosine deaminase ADAR2 allows the site-specific deamination of adenosine to inosine (Cox et al., 2017) providing the first proof-of-principle that this system can be used to site-specifically manipulate mRNAs. Considering that conversion of adenosine to inosine seems to be particularly important for brain development and function (Hwang et al., 2016), this system may provide new avenues to study the role of this modification in neural stem cells and brain development. In addition, it is reasonable to conclude that this approach could readily be adapted to other RNA modifications by fusing the dCas13 with any other relevant RNA-modifying enzyme (Figure 2).

While the CRISPR-dCas13 system proved to be very specific, versatile and efficient it could still harbor potential drawbacks that need to be assessed. For example, the RNA secondary structure may alter binding recognition (Smargon et al., 2017) and therefore limit the available target sites within a transcript. On the other hand, dCas13 binding itself could influence RNA folding, which would be critical while assessing the role of RNA modifications on lncRNAs in which structure underlies function. Finally, although targeting of dCas13 to mRNA seems to not influence translation in general (Cox et al., 2017), it could still affect RNA-protein interactions particularly at regulatory regions or splice-sites resulting in unspecific side-effects. Nevertheless, despite these potential drawbacks, the CRISPR-dCas13 system seems to be broadly applicable to drive various RNA modifications, thus, providing a powerful new tool to filling the gap in knowledge about the molecular function on transcript- and site-specific modifications in functional mRNAs and lncRNAs.

## Conclusions

Although identified decades ago (Desrosiers et al., 1975; Wei et al., 1975), number, abundance, specificity and role of chemical modifications on nucleotide residues of housekeeping and functional RNAs have since remained elusive. As often in science, opening up this new field of epitranscriptomics awaited the development of new methods and technologies that allowed the investigation, for at least a handful of these modifications, of their mechanism of action and physiological role. These breakthroughs led to a number of pioneering studies only in the last few years that clearly pointed toward a regulatory role of epitranscriptome modifications in controlling the stability and metabolism of specific functional RNAs predominantly, although not exclusively, involved in the control of cell fate change and cell type-specific functions.

Among different cell types and tissues, the developing and adult mammalian brain appears to be the organ system more vulnerable to manipulations of the epitranscriptome. For example, although individuals affected by mutations for epitranscriptome writer or eraser genes showed different defects in various organ systems, they all share deficits in brain function including mental retardation and psychological disorders (Boissel et al., 2009; Abbasi-Moheb et al., 2012; Khan et al., 2012; Martinez et al., 2012; Komara et al., 2015). Whether an overall higher vulnerability to any mutation is a general feature of the brain or, alternatively, the epitranscriptome is a relatively late evolutionary addition to the cellular toolkit to attain higher cognitive functions is open to speculation.

With regard to evolution, in the Origin of Species Charles Darwin wrote that natural selection is constantly working to scrutinize *“…the slightest variations; rejecting those that are bad, preserving and adding up all that are good; silently and insensibly working, whenever and wherever opportunity offers, at the improvement of each organic being…”*. In light of this, it is not surprising that the mechanisms that allow the better tuning of gene expression by DNA modifications were revealed to be very similar to the ones used to better tune gene translation by RNA modifications. It is unclear whether during evolution the former were subsequently adapted to attain the latter but given life's origins from an “RNA World” the opposite possibility is also worth considering (Forterre and Grosjean, 2013). Quest for future research will be to decode the specificity and mechanisms underlying the control of RNA modifications and exploit this knowledge by epitranscriptome-editing for basic research and possible applications.

## Author contributions

FN and FC contributed equally to the writing of this manuscript.

### Conflict of interest statement

The authors declare that the research was conducted in the absence of any commercial or financial relationships that could be construed as a potential conflict of interest.
